# A survey on patients' disease perception and the impact of the COVID-19 pandemic on persons living with amyotrophic lateral sclerosis in Malaysia

**DOI:** 10.2217/nmt-2021-0004

**Published:** 2021-07-21

**Authors:** Suzanna Edgar, Nur Adilah Abdul-Aziz, Ee Chin Loh, David Capelle, Khean-Jin Goh, Lydia Abdul Latif, Nortina Shahrizaila, Azlina Ahmad-Annuar

**Affiliations:** ^1^Department of Medicine, Faculty of Medicine, University of Malaya, Kuala Lumpur, 50603, Malaysia; ^2^Department of Biomedical Science, Faculty of Medicine, University of Malaya, Kuala Lumpur, 50603, Malaysia; ^3^Department of Rehabilitation Medicine, Faculty of Medicine, University of Malaya, Kuala Lumpur, 50603, Malaysia

**Keywords:** ALS, COVID-19, disease management, Malaysia, motor neurone disease, survey

## Abstract

**Aim:** To investigate the patients' perception of their disease, its management and the impact of the COVID-19 pandemic on persons living with amyotrophic lateral sclerosis (ALS) in Malaysia. **Patients & methods:** An online survey comprising 42 questions was conducted on ALS patients during the peak of the COVID-19 pandemic. **Results:** Responses were received from 37/60 (62%) participants with ALS directly or through their caregivers. During the COVID-19 pandemic, two-thirds of patients were negatively impacted by the sudden disruption to their hospital appointments, rehabilitation sessions and reduced social interactions. **Conclusion:** This study provided insight into patients' perception of their care and management of ALS in Malaysia which will facilitate in implementing changes that can improve care to persons living with this devastating illness.

Amyotrophic lateral sclerosis (ALS) is a fatal neurodegenerative disease characterized by progressive degeneration of the upper and lower motor neurons resulting in the loss of bulbar function and impaired functional abilities [1]. Death becomes imminent with gradual weakness of the respiratory muscles leading to respiratory failure [2]. Most patients die within 3 years of disease onset, however 10% of patients can exceed 8 years of survival [3].

While there are a number of studies looking at patient experiences in high-income and Western countries, there is limited information about the experiences of ALS patients in low and middle income countries (LMIC) in terms of their understanding of the disease and its management. In LMICs, patients' experiences can vary significantly based on access to trained healthcare professionals, hospital infrastructure and community support from patient advocacy groups. The REPROGRAM Consortium has outlined strategies and recommendations to improve neurodegenerative disease patient management while focusing on LMIC [4].

Due to the relentless progressive nature of the disease, it is critical that patients are referred early to establish the diagnosis and initiate treatment. Historically, communication regarding the disease primarily comes from the clinicians, but with the availability of online resources on ALS, patients and carers are also able to source supporting information to address their concerns, particularly in times of crises such as during the COVID-19 pandemic. The lack of formal supportive care resources in Southeast Asian countries, including Malaysia, often place the responsibility of care on spouses, family members or hired help, who may not fully understand the disease trajectory or best care practices. In addition, cultural advice and marketing strategies regarding use of complementary and alternative medicine (CAM) may complicate their decision making and use of these CAMs may interfere with evidence-based treatment [5]. ALS patients consider the use of at least one type of CAM as an alternative treatment as there is no medically proven therapy to improve their condition. The use of CAMs is largely dependent on the country [6] and common types may include nutritional supplements, traditional medicine (Chinese, Ayurvedic and Malay), acupuncture, stem cell therapy and hydrogen therapy.

Against this backdrop, the current study sought to gather information about the social and medical aspects of persons living with ALS, in particular their use of CAMs and the impact on their finances. In addition, we explored concerns that may have arisen as a result of the COVID-19 pandemic to inform medical providers and support groups on measures that can be implemented to better cater to patients' and caregivers' needs during the ongoing crises where restrictions are placed on movement within the community. At the time of writing, the Motor Neurone Disease (MND) Association, UK, is conducting an online survey to examine the impact of living with this condition during the COVID-19 pandemic [7], thus further highlighting stakeholder interest and urgency in understanding more about this. To the best of our knowledge, this is the first survey in Malaysia, or within Southeast Asia, to investigate similar concerns.

## Patients & methods

ALS patients under active follow-up at University Malaya Medical Centre (UMMC), Kuala Lumpur were invited to participate in an online survey which was conducted over a period of 5 weeks from 28 April to 2 June 2020, coinciding with the Malaysian movement control order. The qualitative questionnaire was designed in a Google form and comprised of 40 questions, provided in both English and Malay language (Supplementary file). Questions were developed using direct statements requiring a response as well as open-ended questions to allow participants to provide further details. The questionnaires were distributed to patients via e-mail and phone messaging links. The study was approved by the UMMC Medical Research Ethics Committee and prior written informed consent was obtained from all patients.

### Statistical analysis

Descriptive statistical analysis was performed using SPSS version 20 to determine the mean, standard deviation, medians, interquartile ranges, frequencies and percentages.

## Results

Sixty patients were invited to participate but only 37 responded either directly or through their caregivers resulting in a response rate of 61.7%. The mean age of participants (n = 22) was 56.6 years (range: 42–74 years) and 81.8% were male. The mean age of caregivers who responded on behalf of patients was 49.1 years (range: 28–75 years) and seven were male. The majority of responses (n = 23; 62.2%) were from patients who had been diagnosed for less than 3 years. The sociodemographic information of respondents is presented in Table 1.

A summary of the key findings from the survey is presented in Table 2.

**Table 1.** Sociodemographic details of the respondents.

Characteristic	Patient n (%)	Caregiver n (%)
Person answering survey	22 (59.5)	15 (40.5)
Age (mean ± SD)	56.6 ± 10.4	49.1 ± 14.0
≤45 years old	5 (22.7)	7 (46.7)
46–60 years old	7 (31.8)	6 (40.0)
>60 years old	10 (45.5)	2 (13.3)
Gender		
Male	18 (81.8)	7 (46.7)
Female	4 (18.2)	8 (53.3)
ALS diagnosis^†^		
≤1 year	14 (37.8)	
2–3 years	9 (24.3)	
>3 years	13 (35.1)	

† Missing data.

ALS: Amyotrophic lateral sclerosis; SD: Standard deviation.

**Table 2.** Summary of key findings from the online survey conducted.

Key issues	n (%)
**Living conditions**	
Living with:	
Spouse/children	31 (83.8)
Relatives/others	2 (5.4)
Live-in nurse	2 (5.4)
Nursing home	2 (5.4)
**Medical and complementary therapy**	
Riluzole medication	11 (29.7)
CAM usage (*accept more than one answer*)	31 (83.8)
Vitamins/supplements	22 (71.0)
Traditional Chinese medicine	15 (48.4)
Malay medicine	4 (12.9)
Ayurvedic treatment	2 (6.5)
Acupuncture	11 (35.5)
Stem cell therapy	2 (6.5)
**Financial impact**	
Patients' decreased earnings after developing ALS^†^	
Yes	23 (62.2)
No	8 (21.6)
Caregivers' decreased earnings after patient developed ALS^†^	
Yes	13 (35.1)
No	12 (32.4)
Caregivers' decreased savings after patient developed ALS^†^	
Yes	20 (54.1)
No	7 (18.9)
Top expenditures related to ALS	
Special food/vitamins	14 (37.8)
Alternative treatments (CAMs)	8 (21.6)
Medication (riluzole)	6 (16.2)
Hired live-in carers	4 (10.8)
**Impact of the COVID-19 pandemic**	
Could continue with physiotherapy session at home	
Yes	9 (24.3)
No	28 (75.7)
Increased stress levels due to the crisis	
Yes	25 (67.6)
No	12 (32.4)
Top reasons for increased stress during the pandemic (*accept more than one answer*)	
Daily routine disrupted	15 (60)
Not knowing what to do during emergency	14 (56)
General medical care interrupted	13 (52)
Do they have a ‘crisis plan’ in place during the restricted movement order if the caregivers become infected	
Yes	6 (16.2)
No	31 (83.8)

† Numbers and percentages are based on 37 responses, except for those with (†), where the patients were given the option to choose not to answer the question, or where more than one answer was accepted.

ALS: Amyotrophic lateral sclerosis; CAM: Complementary and alternative medicine.

### Living conditions & knowledge on ALS

The majority of patients (n = 33; 89.2%) were currently living with their family members, specifically their spouse and/or children or other relatives. Two patients had hired live-in carers, while two other patients had access to day care services. Following their diagnosis, the majority (n = 33; 89.2%) of patients felt they received adequate information about the disease and its progression from their medical providers. Most (n = 31; 83.8%) also sought additional information from the internet, while others contacted the local (n = 13; 35.1%) or international (n = 4; 10.8%) patient support groups or other ALS patients individually (n = 6; 16.2%). Additionally, 35 (95%) of patients valued their local ALS medical team in terms of providing psychological support as well as coordinating training on assistive devices. The local patient advocacy group (MND Malaysia) was also highly valued for peer support, advice on disease management and loaning devices such as noninvasive ventilator (NIV) and eye tracking system.

### Medical & complementary therapy

In terms of ALS medication, only 11 (29.7%) patients were on riluzole. To manage their symptoms, patients relied on rehabilitation (n = 17; 45.9%), noninvasive ventilation (n = 5; 13.5%) and enteral feeding support (n = 3; 8.1%). The majority of patients (n = 31; 83.8%) used at least one form of CAM which included vitamins/supplements, traditional Chinese/Malay medicine, acupuncture and stem cell therapy. Less commonly, Ayurvedic (traditional Indian) treatment and massage were also used to relieve cramps. Interestingly, patients perceived these therapies as being able to slow or stop disease progression (n = 23; 74.2%) and improve overall health (n = 16; 51.6%). Two patients also stated that they believed their disease could be cured by CAMs. One patient indicated that the use of CAMs helped them to adopt a ‘positive mindset’.

### Financial impact

When questioned on the financial impact of their illness, approximately 20% of patients preferred not to respond. The remaining patients reported perceived reductions in their earnings (n = 23; 62.2%) or their caregivers' earnings (n = 13; 35.1%) and savings (n = 20; 54.1%), although detailed financial information was not requested. The biggest financial expenditure was attributed to nutritional support in the form of supplements and enteral feeding supplies (n = 14; 37.8%). Six of the 11 patients on riluzole were paying out-of-pocket. Other expenditures included alternative treatments (n = 8; 21.6%), hired live-in carers (n = 4; 10.8%), medical equipment (n = 3; 8.1%) and medical procedures (n = 2; 5.4%). Other expenses such as daycare fees, cost of traveling to the hospital (from another state), home equipment and renovations were also highlighted. Despite the financial impact, 24 (64.8%) patients did not require personal loan to support the disease management. Almost half (n = 18; 48.6%) had insurance prior to the diagnosis of ALS. However, only 20 (54.1%) were aware that claims could be made based on a diagnosis of ALS under an existing insurance, out of which 13 (65%) were subsequently successful in receiving their claims based on the permanent disability/critical illness clause.

### Living with ALS during the COVID-19 crisis

The majority of respondents (n = 25; 67.6%) reported that the COVID-19 crisis has had a significant impact on their daily lifestyle, causing additional anxiety, largely due to the sudden disruption to their hospital appointments and rehabilitation sessions as well as reduced social activities. Patients were also concerned that there was a need to further rely on others during the pandemic, and expressed their uneasiness that their primary caregiver would not have much respite as living away from other family members meant the caregiving responsibilities could not be shared. Some also expressed the development of agoraphobia due to their underlying condition and were unsure of measures to be taken in the event of an emergency, while others lamented the additional loss of earnings during the pandemic. Only nine patients (24.3%) continued with physiotherapy sessions at home with the help of their caregivers. During the pandemic, 23 (62.2%) patients turned to online resources for ideas and guidance. Of these, ALS/MND related YouTube channels were the most popular (n = 21; 56.8%), followed by other ALS/MND patient support group social media platforms (n = 16; 43.2%) and the MND Malaysia patient support group platform (n = 14; 37.8%). The top searches were for activities and exercises that can be done at home (n = 19; 51.4%), management of medical care in times of crisis (n = 10; 27%) and sourcing out medical supplies (n = 8; 21.6%). When asked about how they would like their consultations to continue if the movement restrictions were extended, the majority of patients (n = 24; 64.9%) preferred phone/video consultation, while 13 (35.1%) would still prefer in-person consultations despite the risks.

## Discussion

In the current study, we found that most ALS patients were happy with the support received from their healthcare providers. It should be noted that the cohort of patients surveyed were recruited from a tertiary center which has a dedicated ALS multidisciplinary team comprising of neurologists, palliative care physicians and rehabilitation physicians. Studies have found that a multidisciplinary approach to care in ALS significantly improves quality of life and survival [8–10]. However, a similar approach may not necessarily be available at other centers in Malaysia where resources are limited. The findings from the current study reinforce the fact that patients require and appreciate a holistic approach to medical care under the management of carefully coordinated services. This is particularly relevant in ALS, which is relatively less common and its symptoms and signs may be overlooked [11]. Diagnostic delays result in failure in implementing early medical intervention as well as potential recruitment into clinical trials.

Currently, there are no effective treatments that can cure or stop the disease progression in ALS. The only US FDA approved drugs, riluzole and edaravone display modest improvement in survival [3]. In the current survey, only a minority of patients (n = 11; 29.7%) were on riluzole. This is likely due to its high cost in Malaysia (USD 420 per month) and the lack of government subsidy. A high percentage (n = 31; 83.8%) opted for CAMs, of which the majority preferred vitamins and supplements, in addition to other types of holistic therapies such as traditional Chinese, Malay and Indian treatments. This is in agreement with studies from other Asian countries including South Korea and China [12,13]. The type and prevalence of alternative therapies are highly dependent on the country [6], whereby the use of vitamins and supplements are widely reported in both Chinese (90.5%) and South Korean (46.7%) ALS patients, while a survey conducted by the German Association for Neuromuscular Diseases reported the wide usage of acupuncture (47%) and homeopathy (40%) [6]. The use of CAMs did represent a significant financial expenditure to patients and caregivers, but they did so with the expectation that these therapies would help with their overall health, although some believed that the treatments could slow or stop disease progression.

As ALS progresses, there is an increased need for ventilatory and nutritional support, both of which have been shown to prolong survival and improve quality of life. However, these measures further add to the financial challenges as shown in our survey. To help with these financial challenges, subsidies are available for some supportive equipment such as NIV through the local patient advocacy group, MND Malaysia, but a recent study on patients from the same center found that despite this provision, not all patients were willing to use NIV [14]. Similar poor acceptance pattern has been observed in other LMIC countries (Korea, China and South Africa) [15–17].

Our patients reported a decline in their financial status since developing ALS. This was largely attributed to the cost of enteral feeding supplies, medical devices and procedures. Similar findings have been reported elsewhere and in Australia, the economic impact of ALS was found to be 1000-times higher than other chronic diseases [18]. Although in many high-income countries, the financial costs are supported by the government but in LMICs, the costs of care are borne by the individual adding to their disease burden. This seemed to be further compounded by the fact that some patients had insurance plans prior to disease onset but few were able to claim for reimbursements due to either a lack of awareness or rejected claims.

We found the majority of our ALS cohort was cared for at home, either by their spouse or other family members. This is similar to what is seen with caregiving for dementia in other Asian communities, for example, Singapore [19] and Taiwan [20], where the decision to place loved ones in nursing facilities is often perceived as a failure of filial piety. Therefore, family members assume the role of caregivers, despite not having the necessary training in handling specialized medical equipment for ALS such as suction machines, ventilators and managing enteral feeding tubes. Given that the bulk of care rests with informal caregivers in Malaysia, there is an urgent need for measures to equip them with adequate skills that will allow caregivers to manage their responsibility of delivering effective care to patients with ALS. In addition, psychological support is also needed to manage the emotional toll that comes with caring for ALS patients. This includes providing options for respite care to caregivers. In our current study, patients and caregivers in our cohort turned to their local patient support group, MND Malaysia for emotional and peer support as well as other international support groups, and these community groups require additional resource in the form of human and financial support to sustain their important outreach activities.

Currently, the COVID-19 pandemic continues to pose a global health risk. Like many countries, Malaysia experienced a surge in cases in early March 2020 [21] which led to the enforcement of stringent public health measures to contain further spread of the virus through movement control order. During this time, only essential activities were permitted and activities involving mass gathering and interstate travel were strictly prohibited. Educational institutions, shopping malls, recreational parks and places of worship were also closed. Routine hospital activities were halted to accommodate COVID-19 cases. ALS patients were considered particularly vulnerable to the infection and the paucity of regular reviews by their healthcare team had an impact on their overall health and wellbeing. Instead, our patients were given the option to have phone consultations over the pandemic period. A recent survey by Glasmacher *et al.* 2020 [22] found that new ALS diagnoses as well as the mortality rate was unaffected by the COVID-19 pandemic in Scotland, largely due to the shielding recommendations implemented by the British government. To our knowledge, a similar policy was not implemented in Malaysia to ‘shield’ the vulnerable population and it is not known if the pandemic affected the diagnosis/mortality rates of ALS in the Malaysian population.

Patients in our study reported a significant disruption to their lifestyle whereby their social life, access to physiotherapy sessions and family members was significantly affected. This in turn caused an increased level of anxiety and stress. During the pandemic, patients found internet resources from ALS social media platforms helpful. The information patients found most helpful were activities and exercises that could be done at home to alleviate some of the complications from their disease.

Importantly for the local ALS community, part of the objective for this survey was to determine the availability of an efficient support system within the patients' homes and immediate community, in the event the main caregiver became ill or was unable to provide care during the pandemic. We found that the majority of respondents were unprepared and did not have a plan on how to manage a prolonged period of restricted medical access. Programs to better prepare patients and their families and to provide ongoing support, virtually or in person is urgently needed and will require input and commitment from all relevant stakeholders.

Our study had several limitations. The survey was performed on patients attending a tertiary ALS center. The same level of care may not be available elsewhere, due to differences in socioeconomic background, geographical limitations and ease of access. Therefore, this survey may not be representative of the entire ALS population of Malaysia particularly those residing in rural areas. The time period for the survey was short as we aimed to capture the challenges posed by the restricted movement order in Malaysia, and a larger future study would help to address the gaps that still remain. The number of respondents was small, but as we chose to offer a degree of anonymity, we could not further query the reasons for the reduced response rate. Despite this, we were still able to get an overview of the management of ALS as perceived by patients including the challenges faced during the COVID-19 pandemic.

## Conclusion

The majority of our patients reported receiving sufficient knowledge on ALS as well as being adequately supported by their local ALS healthcare providers, patient advocacy group and caregivers. However, patients experienced significant financial burden due to the overwhelming cost of managing the disease. The COVID-19 pandemic further added to the anxiety and stress by limiting access to medical and supportive care as well as creating further social isolation. Through understanding these challenges, measures can be implemented to improve the level of support provided to the ALS community especially to patients who continue to suffer from this devastating illness. Given the small number of respondents, which were limited to a single tertiary center, the findings from this survey may not be fully representative of patient experiences in other healthcare settings. Nevertheless, the findings from this survey are useful to inform the community on the needs and challenges faced by ALS patients, and for the need for larger studies.

**Summary points**

- The voice of Malaysian amyotrophic lateral sclerosis (ALS) patients is largely unheard, and there are cultural nuances that need to be taken into consideration in their medical management.
- The majority (n = 33; 89.2%) of Malaysian ALS patients in this cohort were adequately informed about their disease, mainly from their medical providers.
- Complementary and alternative therapies are more commonly used (n = 31; 83.8%) compared with riluzole (n = 11; 29.7%), due to its high cost and lack of government subsidy.
- The disease added to the financial burden by reducing both patients' (n = 23; 62.2%) and their caregivers' earnings (n = 13; 35.1%) and savings (n = 20; 54.1%).
- Government subsidies especially for the US FDA approved drugs riluzole and edavarone may help ease the financial burden of ALS patients.
- Caregivers must be equipped with adequate skills as well as given psychological support to help with caring for ALS patients.
- The recent COVID-19 pandemic revealed their lack of preparedness for crisis management.
- Efforts to provide better support, albeit virtual, from the ALS community will likely help to reduce some of the anxiety and social isolation experienced by patients and their caregivers during the pandemic.
