# Supplementary material for: A survey on patients' disease perception and the impact of the COVID-19 pandemic on persons living with amyotrophic lateral sclerosis in Malaysia
Source: Neurodegener Dis Manag. 2021 Jul 21:10.2217/nmt-2021-0004. doi: 10.2217/nmt-2021-0004 (PMC8320654; doi:10.2217/nmt-2021-0004)
Supplement: Supplementary file 1 [file supplementary_file.pdf]

Motor Neurone Disease Survey (management of care, financial implications, dealing with crisis, support system) / Tinjauan bagi penyakit Motor Neuron (pengurusan penjagaan, implikasi kewangan, menangani krisis, sistem sokongan)

The Motor Neurone Disease (MND) team at University Malaya Medical Centre and the Faculty of Medicine, University of Malaya is conducting a survey about your experience living with MND. It covers 4 main areas (your understanding of the disease, your MND care, any effects on your finances), as well as a section on whether the lockdown due to the COVID19 pandemic has had any effect on your or your caregivers' lives.
The information will help us better understand how you are coping as an MND patient, and help to inform others and us about your opinion on MND. Some of the information may be shared at medical meetings or forums related to MND because your opinion is very valuable to the MND community in Malaysia and across the world.
This is a voluntary survey. You do not have to participate if you do not want to. We will not ask for any personal information. We are grateful for your participation and hope you can give us your honest feedback. The survey will only take about 10 -15mins to answer.
If you have any queries about this survey, please contact Professor Dr. Nortina Shahrizaila, Consultant Neurologist, University Malaya Medical Centre (nortina@ummc.edu.my).

Pasukan Penyakit Motor Neuron (MND) di Pusat Perubatan Universiti Malaya dan Fakulti Perubatan Universiti Malaya sedang menjalankan tinjauan mengenai pengalaman anda yang menghidap MND. Tinjauan ini merangkumi 4 bidang utama (pemahaman anda mengenai penyakit ini, penjagaan MND anda, kesan penyakit ini terhadap kewangan anda) serta bahagian mengenai kesan pandemik COVID19 dan perintah kawalan pergerakan (PKP) terhadap kehidupan anda atau penjaga anda.
Maklumat tersebut akan membantu pihak kami memahami dengan lebih baik bagaimana anda mengendalikan kehidupan anda sebagai seorang pesakit MND, dan pendapat anda tentang penyakit ini. Maklumat daripada tinjauan ini mungkin dikongsikan di mesyuarat dan forum berkaitan dengan MND kerana pendapat dan pengalaman anda sangatlah berharga kepada komuniti MND di Malaysia dan seluruh dunia.
Penyertaan anda dalam tinjauan ini adalah secara sukarela, dan kami tidak meminta butiran pengenalan peribadi. Kami sangatlah berterima kasih di atas penyertaan anda dan berharap anda dapat memberikan maklumbalas yang jujur. Tinjauan ini akan mengambil 10-15min sahaja untuk menjawabnya.
Sekiranya anda mempunyai pertanyaan mengenai tinjauan ini, sila hubungi Profesor Dr. Nortina Shahrizaila, Pakar Perunding Neurologi, PPUM (nortina@ummc.edu.my).

* Required / Diperlukan

1. Email address *

2. Age / Umur *

3. Gender / Jantina *

Mark only one oval.

- Male / Lelaki
- Female / Perempuan

4. Person answering this survey is the: / Pihak yang menjawab tinjauan ini adalah: *

Mark only one oval.

- Patient / Pesakit
- Caregiver / Penjaga

About your MND diagnosis / Mengenai diagnosis MND anda

5. Year of MND diagnosis / Tahun diberi diagnosis MND *

6. Following your diagnosis, were you given enough information about the disease and how it would progress? / Setelah diberi diagnosis, adakah anda diberikan maklumat yang mencukupi mengenai penyakit ini dan bagaimana perkembangan lanjutan penyakit ini? *

Mark only one oval.

- Yes / Ya
- No / Tidak

7. If you answered no, would you have wanted more information from the doctor? / Sekiranya anda menjawab tidak, adakah anda ingin menerima maklumat yang lebih mendalam daripada doktor anda?

Mark only one oval.

- ◯ Yes / Ya
- ◯ No / Tidak

8. Where do you find more information about MND? / Di mana anda mendapat lebih banyak maklumat mengenai MND ini? *

Tick all that apply / Tandakan semua yang berkaitan.

- ☐ Local support groups / Kumpulan sokongan pesakit MND tempatan
- ☐ International support groups / Kumpulan sokongan pesakit MND antarabangsa
- ☐ Information from internet / Maklumat daripada internet
- ☐ Other MND patients / Pesakit MND lain

9. What treatment are you currently taking? / Rawatan manakah sedang anda ambil sekarang?

Tick all that apply / Tandakan semua yang berkaitan

- ☐ Riluzole / Riluzole
- ☐ Rehab support / Sokongan rehab
- ☐ Non-invasive ventilation
- ☐ Gastrostomy tube
- ☐ Alternative therapy / Terapi alternatif

10. If you are taking alternative therapies, which of these are you currently using? / Jika anda sedang mengamalkan terapi alternatif, adakah anda menggunakan kaedah berikut?

Tick all that apply / Tandakan semua yang berkaitan

- ☐ Vitamins/Supplements / Vitamin/Nutrient tambahan
- ☐ Stem cell therapy / Terapi sel stem
- ☐ Acupuncture / Akupuntur
- ☐ Hydrogen therapy / Terapi hidrogen
- ☐ Traditional Chinese Medicine / Perubatan tradisional Cina
- ☐ Malay medicine (e.g: Habbatus sauda) / Perubatan tradisional Melayu (cth: Habbatus sauda)

11. How do you think the alternative therapies might help? / Bagaimana anda fikir terapi alternatif ini boleh membantu?

Tick all that apply / Tandakan semua yang berkenaan

Tick all that apply / Tandakan semua yang berkaitan

- ☐ Cure / Penawar
- ☐ Slow down/stop the progression / Melambatkan/menghentikan progres penyakit MND
- ☐ Improve your MND symptoms / Memperbaiki simptom penyakit MND anda
- ☐ Improve your overall health / Meningkatkan kesihatan badan anda secara keseluruhan

12. Others (state) / Lain-lain (nyatakan):

____

____

____

____

____

About your care / Mengenai penjagaan anda

13. Currently, who do you live with? / Pada masa ini, anda sedang tinggal bersama siapa? *

Tick all that apply / Tandakan semua yang berkaitan

- [ ] Alone / Sendiri
- [ ] Spouse / Pasangan
- [ ] Children / Anak
- [ ] Relative / Saudara
- [ ] Hired live-in Nurse/Helper / Jururawat/Pembantu yang diupah
- [ ] Nursing home / Pusat jagaan
- [ ] Others / Lain-lain

14. Who is your primary carer? / Siapakah penjaga utama anda? *

____

15. Do you feel supported by your MND medical team? / Adakah anda berasa anda mendapat sokongan yang mencukupi daripada pasukan perubatan MND anda? *

Mark only one oval.

- ( ) Not at all / Tidak sama sekali
- ( ) In some aspects / Dalam beberapa aspek
- ( ) In every aspect / Dalam setiap aspek

16. Please provide an explanation for your answer in no. 10 / Sila jelaskan jawapan anda di no. 10.

____

____

____

____

____

17. Do you feel supported by a local patient support group (e.g: MND Malaysia)? / Adakah anda berasa anda mendapat sokongan yang mencukupi daripada kumpulan sokongan pesakit MND tempatan (cth: Persatuan MND Malaysia)? *

Mark only one oval.

- Not at all / Tidak sama sekali
- In some aspects / Dalam beberapa aspek
- In many aspects / Dalam banyak aspek
- In every aspect / Dalam setiap aspek

18. Which particular means of support have been most meaningful to you and your caregiver? / Sokongan yang manakah paling bermakna kepada anda dan penjaga anda? *

Has MND affected your financial situation? / Adakah MND mempengaruhi situasi kewangan anda?

19. My earnings have significantly decreased after I developed MND / Pendapatan saya telah menurun dengan ketara setelah saya menghidapi MND *

Mark only one oval.

- Yes / Ya
- No / Tidak
- Prefer not to answer / Pilih untuk tidak menjawab

20. My caregivers’ earnings have significantly decreased after I developed MND / Pendapatan penjaga saya telah menurun dengan ketara setelah saya menghidapi MND *

Mark only one oval.

- Yes / Ya
- No / Tidak
- Don't know / Tidak tahu
- Prefer not to answer / Pilih untuk tidak menjawab

21. My / my caregivers' savings have significantly decreased after I developed MND / Simpanan saya / penjaga saya telah berkurang dengan ketara setelah saya menghidapi MND *

Mark only one oval.

- Yes / Ya
- No / Tidak
- Prefer not to answer / Pilih untuk tidak menjawab

22. What has been the biggest financial burden? / Apakah beban kewangan yang paling besar? *

Mark only one oval.

- Home renovation / Pengubahsuaian rumah
- Hired care / Upah pengasuh
- Equipment / Peralatan perubatan
- Medication (Riluzole, Edaravone)/ Ubat (Riluzole, Edaravone)
- Alternative treatments / Terapi alternatif
- Special food / Vitamin/nutrien tambahan
- Medical procedures / Prosedur perubatan

23. Others (state) / Lain-lain (nyatakan)

____

24. Did you have insurance before your MND diagnosis? / Adakah anda mempunyai insurans sebelum diagnosis MND anda? *

Mark only one oval.

- ( ) Yes / Ya
- ( ) No / Tidak

25. Are you aware that a diagnosis of MND can be claimed under an existing insurance? / Adakah anda sedar bahawa diagnosis MND boleh dituntut berdasarkan polisi insurans yang ada? *

Mark only one oval.

- ( ) Yes / Ya
- ( ) No / Tidak

26. If you answered yes, have you been able to get insurance claims based on permanent disability/critical illness? / Sekiranya anda menjawab ya, adakah anda berjaya mendapat tuntutan insurans berdasarkan ketidakupayaan kekal / penyakit kritikal?

Mark only one oval.

- ( ) Yes / Ya
- ( ) No / Tidak

27. To what extent has the COVID19 crisis changed your daily life? / Sejauh mana krisis COVID19 mengubah kehidupan harian anda? *

Mark only one oval.

- Not at all / Tidak sama sekali
- Hardly / Hampir tidak
- For some matters / Untuk beberapa perkara
- Rather disruptive / Agak mengganggu
- Very disruptive / Sangat mengganggu

28. What has changed? / Apa yang telah berubah?

29. During this time, which type of consultation would you have liked? / Di sepanjang tempoh ini, apakah jenis konsultasi yang anda inginkan? *

Mark only one oval.

- In-person (ie still able to attend clinic) / secara peribadi (cth masih membuat lawatan ke klinik)
- Video consultation / Konsultasi melalui video
- Phone consultation / Konsultasi melalui talian

30. Other (please state) / Lain-lain (sila nyatakan)

31. Are you still receiving physiotherapy at home? / Adakah anda masih menerima terapi fisio di rumah?

Mark only one oval.

- Yes / Ya
- No / Tidak

32. Has the crisis increased your stress levels? / Adakah krisis ini telah meningkatkan tahap kerisauan anda? *

Mark only one oval.

- Yes / Ya
- No / Tidak

33. If you answered yes, rate what are the things that made you feel stressed? / Sekiranya anda menjawab ya, pilih perkara-perkara yang mengakibatkan anda berasa tertekan/risau? *

Tick all that apply / Tandakan semua yang berkaitan

- [ ] Interruptions to general medical care / Gangguan dalam rawatan perubatan am
- [ ] Interruptions to essential medical supplies / Gangguan bekalan perubatan penting
- [ ] Not knowing what to do if you have an emergency (since the hospitals are full of COVID19 cases) / Tidak tahu apa yang harus dilakukan sekiranya menghadapi kecemasan (kerana hospital penuh dengan kes COVID19)
- [ ] Disruption to your usual routine / Gangguan pada rutin biasa anda
- [ ] Crucial visitors/packages may bring the virus into the house / Pelawat/bungkusan parcel boleh membawa virus ke dalam rumah
- [ ] Having to rely more on others / Harus bergantung lebih pada orang lain
- [ ] Financial worries / Bimbang mengenai kewangan
- [ ] Stress on caregivers / Lebih tekanan ke atas penjaga
- [ ] Loss of contact with friends/community/visitors / Hilang hubungan dengan rakan/komuniti/pelawat
- [ ] Crowded feeling because there are more people in the house / Rasa sesak kerana terdapat lebih ramai orang di rumah

34. Others (state) / Lain-lain (nyatakan)

35. Have you looked up any online resources for additional info about ALS/MND during this period? / Adakah anda mencari maklumat tambahan mengenai ALS/MND melalui internet semasa tempoh ini?

Mark only one oval.

- ◯ Yes / Ya
- ◯ No / Tidak

36. If you answered yes, what resources have you used? / Sekiranya anda menjawab ya, pilih sumber yang telah digunakan? *

Tickk all that apply / Tandakan semua yang berkaitan

- ☐ MND Malaysia patient support group Facebook or chat group / Facebook atau 'chatgroup' kumpulan sokongan pesakit MND Malaysia
- ☐ Other MND/ALS patient support groups Facebook or websites / Facebook atau laman web kumpulan sokongan lain untuk pesakit MND/ALS
- ☐ World Health Organisation (WHO) / Organisasi kesihatan dunia (WHO)
- ☐ MND/ALS related Youtube channels or social media accounts / Saluran Youtube atau akaun sosial media yang berkaitan MND/ALS

37. Others (state) / Lain-lain (nyatakan)

38. What information has been most helpful? / Maklumat yang manakah yang paling berguna? *

Mark only one oval.

- ( ) Related to management of medical care in times of crisis / Berkaitan dengan pengurusan rawatan perubatan semasa krisis
- ( ) Suggestions of activities/exercises that can be done at home / Cadangan aktiviti/latihan yang boleh dilakukan di rumah
- ( ) Where to find medical supplies / Mencari bekalan perubatan

39. Others (specify) / Lain-lain (nyatakan)

____

40. Do you and your caregivers have a 'crisis plan' about what to do if the COVID19 restricted movement order continues for several months/if your main caregivers get infected with the virus? / Adakah anda atau penjaga anda mempunyai 'rancangan krisis' tentang apa yang harus dilakukan sekiranya perintah kawalan pergerakan COVID19 berlanjutan selama beberapa bulan/ jika penjaga utama anda dijangkiti dengan virus tersebut? *

Mark only one oval.

- ( ) Yes / Ya
- ( ) No / Tidak

41. If you answered yes, what is the plan? / Sekiranya anda menjawab ya, apakah rancangan tersebut?

____
____
____
____
____

42. Please add any further comments for issues that may not have been addressed in the questions above / Sila tambah sebarang komen untuk isu yang tidak diliputi dalam soalan-soalan di atas.

Thank you very much for participating in this survey. / Terima kasih di atas penglibatan anda dalam tinjauan ini.

Best wishes from the UMMC MND team. / Salam sejahtera daripada pasukan MND PPUM.

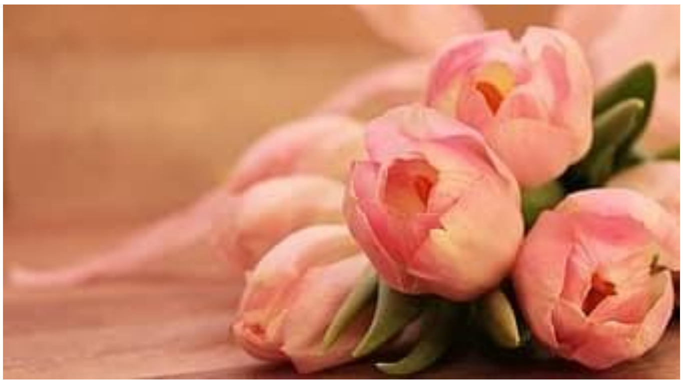

This content is neither created nor endorsed by Google.

Google Forms
